# Identification of Pathogenic Variant Burden and Selection of Optimal Diagnostic Method Is a Way to Improve Carrier Screening for Autosomal Recessive Diseases

**DOI:** 10.3390/jpm12071132

**Published:** 2022-07-12

**Authors:** Evgeniia A. Sotnikova, Anna V. Kiseleva, Vladimir A. Kutsenko, Anastasia A. Zharikova, Vasily E. Ramensky, Mikhail G. Divashuk, Yuri V. Vyatkin, Marina V. Klimushina, Alexandra I. Ershova, Karina Z. Revazyan, Olga P. Skirko, Marija Zaicenoka, Irina A. Efimova, Maria S. Pokrovskaya, Oksana V. Kopylova, Anush M. Glechan, Svetlana A. Shalnova, Alexey N. Meshkov, Oxana M. Drapkina

**Affiliations:** 1 National Medical Research Center for Therapy and Preventive Medicine, Ministry of Healthcare of the Russian Federation, Petroverigsky per.10, Bld. 3, 101000 Moscow, Russia; sotnikova.evgeniya@gmail.com (E.A.S.); vlakutsenko@yandex.ru (V.A.K.); azharikova89@gmail.com (A.A.Z.); ramensky@gmail.com (V.E.R.); divashuk@gmail.com (M.G.D.); vyatkin@gmail.com (Y.V.V.); mklimushina@gmail.com (M.V.K.); alersh@mail.ru (A.I.E.); mdmuradyan@bk.ru (K.Z.R.); ops_70@mail.ru (O.P.S.); biobank@gnicpm.ru (I.A.E.); mpokrovskaia@list.ru (M.S.P.); sivoksana@yandex.ru (O.V.K.); aglechan@gnicpm.ru (A.M.G.); sshalnova@gnicpm.ru (S.A.S.); meshkov@lipidclinic.ru (A.N.M.); drapkina@bk.ru (O.M.D.); 2 Faculty of Mechanics and Mathematics, Lomonosov Moscow State University, 1-73, Leninskie Gory, 119991 Moscow, Russia; 3 Faculty of Bioengineering and Bioinformatics, Lomonosov Moscow State University, 1-73, Leninskie Gory, 119991 Moscow, Russia; 4 All-Russia Research Institute of Agricultural Biotechnology, Timiryazevskaya Street, 42, 127550 Moscow, Russia; 5 Novosibirsk State University, 1, Pirogova Str., 630090 Novosibirsk, Russia; 6 Moscow Institute of Physics and Technology, Dolgoprudny, Institutskiy per.9, 141701 Dolgoprudny, Russia; marija.zaicenoka@gmail.com

**Keywords:** carrier screening, *CFTR*, *PAH*, *SERPINA1*, *GJB2*, autosomal recessive disorders, population, allele frequency

## Abstract

Cystic fibrosis, phenylketonuria, alpha-1 antitrypsin deficiency, and sensorineural hearing loss are among the most common autosomal recessive diseases, which require carrier screening. The evaluation of population allele frequencies (AF) of pathogenic variants in genes associated with these conditions and the choice of the best genotyping method are the necessary steps toward development and practical implementation of carrier-screening programs. We performed custom panel genotyping of 3821 unrelated participants from two Russian population representative samples and three patient groups using real-time polymerase chain reaction (PCR) and next generation sequencing (NGS). The custom panel included 115 known pathogenic variants in the *CFTR*, *PAH*, *SERPINA1*, and *GJB2* genes. Overall, 38 variants were detected. The comparison of genotyping platforms revealed the following advantages of real-time PCR: relatively low cost, simple genotyping data analysis, and easier detection of large indels, while NGS showed better accuracy of variants identification and capability for detection of additional pathogenic variants in adjacent regions. A total of 23 variants had significant differences in estimated AF comparing with non-Finnish Europeans from gnomAD. This study provides new AF data for variants associated with the studied disorders and the comparison of genotyping methods for carrier screening.

## 1. Introduction

Carrier screening is the genetic testing of unaffected individuals for the purpose of identifying those who have one allele associated with an autosomal recessive disorder. Thus, subsequently it helps to detect couples with a one-in-four chance of having an affected child. Those couples should be provided with information that can influence their reproductive decision [1]. It has been shown that implementation of carrier screening may result in the reduction in the number of new disease cases up to 97% [2]. While expanded carrier screening with NGS is becoming more common, targeted testing approach remains a valid detection tool for the most widely distributed deleterious variants [3], especially in the case of variants, which account for a major part of the disease occurrence. There are no carrier screening programs in Russia, and the majority of the available panels [4,5,6] detect only a few variants most common for the disease. Besides there are only a limited number of studies with Russian population-based allele frequency (AF) data [6,7,8,9,10].

The conditions chosen in this study for carrier screening in Russia are common autosomal recessive disorders that have a well-defined phenotype and a detrimental effect on life expectancy and/or quality: cystic fibrosis (CF, OMIM #219700), phenylketonuria (PKU, OMIM #261600), alpha-1 antitrypsin deficiency (A1ATD, OMIM #613490), and sensorineural hearing loss (SNHL, OMIM #220290). Using neonatal screening, 1579 children with CF and 4425 with PKU were identified in Russia for the period from 2008 to 2020 [11]. CF is caused by mutations in the *CFTR* gene, affecting organ systems containing epithelia and resulting in severe decline in living standard and life expectancy [12]. PKU is characterized by a decreased catalytic activity of phenylalanine hydroxylase that results predominantly from mutations in the *PAH* gene. In patients without treatment, PKU leads to severe intellectual disability beginning within the first few months of life [13].

The most common cause of SNHL is the recessive mutations in the *GJB2* gene, which encodes protein connexin 26, a structural component of the intercellular channels [14]. The prevalence of SNHL associated with *GJB2* variants is 1:1000, and every 16th Russian is a carrier of *GJB2* variants [14]. 

A1ATD originates from mutations in the serpin peptidase inhibitor clade A gene (*SERPINA1*) and has diverse clinical presentations from asymptomatic to fatal liver or lung disease [15]. The most common pathogenic alleles are PiS (rs17580) and PiZ (rs28929474), and their presence leads to reduced expression level of alpha-1 antitrypsin (up to 50–60% and 10–20%, respectively) [16]. The study based on the UK Biobank data discovered a high rate of A1ATD underdiagnosis; only 6.4% of the participants with PI*ZZ genotypes were diagnosed with A1ATD [17]. Blanco et al. combined the results of five studies that published AF of these variants in Russia and found 10/1000 for PiS and 3/1000 for PiZ [18]. According to the Russian Ministry of Health Care, there were no epidemiological studies on the prevalence of A1ATD in Russia [19].

To find a balance between the size of the panel (and therefore cost of screening) and the proportion of carriers it allows to detect, it is necessary to know the AF in the target population [6]. We designed a custom panel for carrier screening of the four aforementioned diseases consisting of 115 variants, which occur in the Russian population according to the literature data [4,20,21,22,23,24,25,26]. This custom panel was validated on the population-based sample, representing 1243 unrelated individuals from the Vologda region [8], as well on 350 volunteers interested in carrier screening. The TaqMan real-time polymerase chain reaction (PCR) platform was used for this part of the study since it allows fast genotyping of a relatively large number of variants for a group of samples. However, subsequent validation revealed some issues concerning accuracy of genotyping; therefore, we reexamined the same set of variants on a different platform and collected more validation data. Next generation sequencing (NGS) was chosen as the most comprehensive diagnostic tool for genotyping that also allows the identification of some additional variants in adjacent regions. The NGS custom panel included the same 115 variants of genes (*CFTR*, *PAH*, *SERPINA1*, *GJB2*) associated with CF, PKU, A1ATD, and SNHL, respectively, and was tested on a representative sample from another region (Ivanovo) [9] with a close ethnic background (95.57% Russians) [27].

The aim of this study was to evaluate AF for 115 variants in *CFTR*, *PAH*, *SERPINA1*, and *GJB2* genes based on two population samples from regions with a close ethnic composition and to compare obtained AF with known European data. Herein, we represent the results of the NGS sequencing and TaqMan genotyping of 3821 unrelated participants with further comparison of both genotyping methods. This new information could expand known population-based data for CF, PKU, A1ATD, and SNHL in the Russian population and can be used for carrier-screening programs.

## 2. Materials and Methods

### 2.1. Selection of Participants and Clinical Data

We studied participants from five large groups, as described below (Table 1).

Two population samples of Ivanovo and Vologda regions, collected for the cross-sectional study “Epidemiology of Cardiovascular Diseases and Risk Factors in Regions of the Russian Federation” (ESSE) [28] were analyzed in this research, which were the representative samples of ESSE-Ivanovo (*n* = 1858) [9] and ESSE-Vologda (*n* = 1244) [8]. After quality control, PCA, and estimation of relatedness, 192 individuals were excluded from analysis. Thus, data from 1667 participants of ESSE-Ivanovo (median age was 49 years old (39; 56); 37.1% were men) and 1243 of ESSE-Vologda (median age was 45 years old (34; 54); 46.1% were men) were used for AF calculation. 

Bauman Center sample (BCS) was formed based on the carrier screening study that included 535 clinic visitors of reproductive age of *n*.E. Bauman Scientific and educational medical-technological center (median age was 19 years old (18; 21); 59.5% were men), the biological samples were obtained for 429 of them. Only genotyped participants with call rate above 90% were included in the analysis. Data from a total of 350 individuals were used for AF calculation.

Russian patient sample (RPS) consisted of data from custom panel (RPS-CP, *n* = 539) and exome (RPS-E, *n* = 194) sequencing and were formed of patients observed at the National Medical Research Center (NMRC) for Therapy and Preventive Medicine (Moscow, Russia) with various medical conditions. A total of 172 individuals (99 from RPS-CP and 73 from RPS-E) were excluded from analysis after quality control, PCA, and estimation of relatedness. Data from a total of 440 from RPS-CP individuals (median age was 47 years old (36; 59); 48% were men) and 121 from RPS-E (median age was 48 years old (36; 58); 52.9% were men) were used for AF calculation of 115 variants. 

The storage of all blood samples and buccal swabs (only for BCS) was performed at −30 °C and +4 °C, respectively, at the Biobank of the NMRC for Therapy and Preventive Medicine (Moscow, Russia).

Overall, genotyping analysis was performed for 4264 participants, and AF calculation data from 3821 participants were used. The clinical data were collected from questionnaires of the NMRC for Therapy and Preventive Medicine (Moscow, Russia) and of the ESSE-RF study (2012).

### 2.2. DNA Extraction

Genomic DNA was extracted from peripheral blood or buccal swab samples with the use of QIAamp DNA Blood Mini Kit (Qiagen, Hilden, Germany). Qubit 4.0 fluorimeter (Thermo Fisher Scientific, Waltham, MA, USA) or NanoDrop OneC spectrophotometer (Thermo Fisher Scientific, Waltham, MA, USA) were used for measuring the DNA concentration.

### 2.3. Custom Panel

The TaqMan custom panel used in this study was described previously [8]. The NGS custom panel was created on its basis and consisted of the same 115 variants (Supplementary Table S1). The TaqMan custom panel included 116 TaqMan assays because one large deletion CFTRdele2,3 (hg19::chr7:117138367-117159446) required two assays for correct detection (Supplementary Table S2). Among 115 variants, 65 were in *CFTR*, including four multiallelic variants (rs121908746, rs121908751, rs121908805, rs77932196), 23 in *PAH*, 10 in *SERPINA1*, and 17 in *GJB2* gene.

### 2.4. Real-Time PCR

Genotyping of 115 variants using 116 Taqman assays in the *CFTR*, *PAH*, *SERPINA1*, and *GJB2* genes in ESSE-Vologda and BCS was performed by real-time PCR using QuantStudio 12 K Flex (Thermo Fisher Scientific, Waltham, MA, USA) according to the manufacturer’s protocols as described previously [8,21,22]. The average accuracy of genotyping, the call rate using the QuantStudio 12 K Flex Real-Time PCR system, was 94.2% for ESSE-Vologda and 97.5% for BCS.

### 2.5. Next Generation Sequencing

Genotyping of 115 variants in ESSE-Ivanovo and RPS was performed by NGS. The libraries for the NGS custom panel were prepared using the SeqCap EZ Prime Choice Library kit (Roche, Basel, Switzerland). Exome libraries were prepared using IDT-Illumina TruSeq DNA Exome protocol (Illumina, San Diego, CA, USA). Sequencing was performed on a Nextseq 550 (Illumina, San Diego, CA, USA). All sequencing stages were performed according to the manufacturers’ protocols.

### 2.6. Sanger Sequencing

The validation of results by Sanger sequencing was performed for selected samples of ESSE-Vologda, ESSE-Ivanovo, RPS, and all samples of BCS with detected deleterious variants. Sanger sequencing was performed on DNA sequencer Applied Biosystem 3500 DNA Analyzer (Thermo Fisher Scientific, Waltham, MA, USA) using the ABI PRISM BigDye Terminator v3.1 reagent kit (Thermo Fisher Scientific, Waltham, MA, USA) according to the manufacturer’s protocol.

### 2.7. Bioinformatic Processing of NGS Data

All bioinformatic analyses were described in more detail in the previous study of the ESSE-Ivanovo sample [9]. Sequencing analysis resulted in fastq files; paired-end reads were aligned to the GRCh37 reference genome. Data processing and quality control evaluation were performed with the custom-designed pipeline based on GATK 3.8 [29]; in particular, we used GENOTYPE_GIVEN_ALLELES mode of GATK HaplotypeCaller for genotyping. GATK hard filters flagged all detected variants as PASS; among all 115 variants, GQ values were maximal (GQ = 99) for 97.66% (custom panel) and 97.85% (exome) genotypes. The annotation of single-nucleotide variants and short indels was performed with ClinVar (2021/01/10) [30], gnomAD (v2.1.1) databases [31], and dbSNP [32] databases. 

PLINK v1.90 [33] was used for NGS data to obtain identity by state (IBS) values and identity by descent (IBD) proportion (PI_HAT) for all pairs of individuals. To ensure our dataset does not contain closely related individuals, we removed a younger participant from each pair with PI_HAT > 0.33. Then, the PCA of individual genotypes was performed by HWE-normalized PCA analysis using Hail library v.0.2.83-b3151b4c4271 [34]. We excluded from PCA analysis variants with minor AF < 5% and performed linkage disequilibrium pruning with R^2^ = 0.2. The PCA was performed separately for samples sequenced using custom panel and exomes; the final set of variants analyzed included 2272 variants from custom panel and 22,678 variants from exome. 

Copy number variation (CNV) analysis for detecting large indels (e.g., CFTRdele2,3) was performed using CNVkit2 with default parameters using circular binary segmentation [35] and Haar method for the segmentation [36]. CNV analysis was conducted only for *CFTR* and *PAH*, because *SERPINA1* and *GJB2* genes were not covered sufficiently in the custom panel to reliably determine the CNV.

### 2.8. Statistical Analysis of Variant Frequencies

All statistical analyses were conducted using R v. 4.1.2 (R Foundation for Statistical Computing, Vienna, Austria) [37]. The age was presented using median and interquartile range. The comparison of AF between groups of participants was performed by Fisher’s exact test. Combining variants by genes was performed by assigning “1” to the participant if there was at least one variant in the gene and “0” otherwise. The comparison of the AF with the reference ones in the gnomAD NFE population was performed using a binomial test. The correction for multiple comparisons was performed using the Benjamini–Hochberg procedure (p adj). The Clopper–Pearson exact method was used for estimation of 95% confidence interval. The Hardy–Weinberg equilibrium was tested using an exact test. We judged associations statistically significant if the *p* value was less than 0.05.

## 3. Results

### 3.1. Population Substructure Analysis

**Figure 1:**
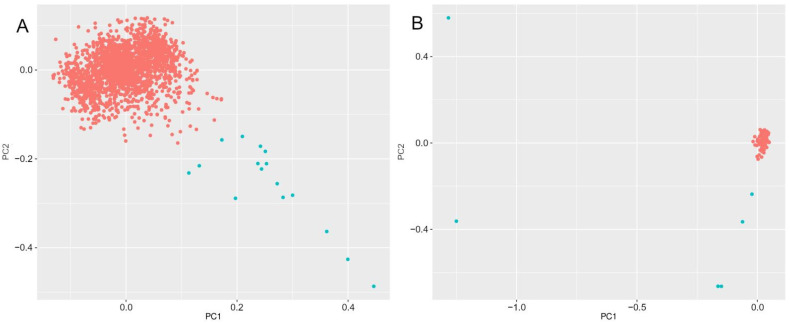
Principal component analysis of the substructure of the studied population: (**A**) ESSE-Ivanovo and RPS-CP; and (**B**) RPS-E. The core cluster is colored red, and outliers are colored blue.

Analysis of the fine genetic structure of the studied population was conducted using standard principal component analysis (PCA) plot procedure (Figure 1). We removed 22 outlier samples that apparently did not represent the major Russian ethnic group that comprises the dense core of the plot: 4 from ESSE-Ivanovo and 12 from RPS-CP (Figure 1A) and 6 from RPS-E (Figure 1B). Among 22 removed samples, we found one allele of rs5030858 in the *PAH* gene but did not include it in AF calculation due to the small size of the outlier group.

### 3.2. Genotyping Analysis

Overall, genotyping analysis detected 38 variants from 115 studied ones (Supplementary Table S3). There were no deviations from the Hardy–Weinberg equilibrium for all variants, including the most frequent ones rs35887622 and rs28929474, except for rs80338939. It significantly deviated from Hardy–Weinberg equilibrium in RPS-E (*p* = 0.025). Table 2 contains the counts of the detected variants in the studied groups of samples. The number of carriers was estimated as the number of participants carrying at least one alternative allele. The most frequent variants (above 1% in each of population samples) were rs35887622 (*GJB2*) and rs28929474 (*SERPINA1*). Additionally, rs17580 (*SERPINA1*) was observed with AF of 1.35% in ESSE-Vologda and 0.93% in ESSE-Ivanovo and with rs80338939 (*GJB2*) with AF of 1.74% in ESSE-Ivanovo (it was not detected in ESSE-Vologda due to the technical issues). On the other end of the frequency spectrum, eight variants were detected only once across all samples: rs80338950 (*GJB2*), rs5030860, rs76296470, rs5030843 (*PAH*), rs397508612, rs80034486, rs75039782, and rs75541969 (*CFTR*). 

We observed no statistically significant differences in AF between two population samples after Benjamini–Hochberg adjustment (Supplementary Table S4). This result agrees with a close resemblance of ethnic composition of these regions. Carriers of rs80338939 (*GJB2*) are presented in Table 2 but were not considered for carrier percentage comparison between population samples.

Nine participants carried two variants in one gene: five in ESSE-Ivanovo, one in RPS-E, two in ESSE-Vologda, and one in BCS (Supplementary Table S5). In four cases, participants were homozygous, and in two other cases, due to the close proximity of variants, we were able to confirm compound heterozygosity. The available clinical data did not contain information that could confirm the studied disorders. A total of 35 individuals carried two variants in different genes: 15 in ESSE-Ivanovo, ten in RPS-CP, nine in ESSE-Vologda, and one in BCS.

Besides the included variants NGS allows to identify 15 carriers of 13 additional pathogenic or likely pathogenic variants in adjacent sequenced regions (Table 3, Supplementary Table S6), the Clinvar pathogenicity data was used [30]. Combining these data with genotyping results for 115 variants included in the custom panel, it was found that two of them had two pathogenic variants in the studied genes: rs17580 in *SERPINA1* and rs62507344 in *PAH*; and rs17580 in *SERPINA1* and rs542645236 in *PAH*.

### 3.3. Differences in Allele Frequencies between Studied Samples and Non-Finnish Europeans and Closely Related Population Data

We compared obtained AF with those reported for the non-Finnish Europeans (NFE) in the gnomAD database [31]. A total of 17 variants out of 38 observed in our study had statistically significant differences in AF between our data and gnomAD data (Table 4). For 15 of them, the differences were significant for ESSE-Ivanovo and ESSE-Vologda together (Supplementary Table S7), and for the remaining two variants, the difference was significant only for the combined data from all the samples included in this study (Supplementary Table S8). AF of five variants (rs17580, rs28929474, rs28931570 (*SERPINA1*); rs113993960, rs78655421 (*CFTR*)) were significantly higher in the NFE, and the remaining 12 were significantly higher in the Russian population.

Analysis of the detected additional pathogenic and likely pathogenic variants in the adjacent sequenced regions revealed four variants in population ESSE-Ivanovo sample (Supplementary Table S9), two in RPS-E, and one in RPS-CP (for combined AF for RPS-CP and ESSE-Ivanovo) with AF significantly higher than in the gnomAD NFE population (Table 5, Supplementary Table S10).

Furthermore, we performed a comparison of the obtained AF with those from recently published data for the Russian population [6,10]. There were no statistically significant differences in AF between our results and these two population studies, which is consistent with a close ethnic composition of studied groups of samples. Results are presented in Supplementary Table S11 for the custom panel and in Supplementary Table S12 for additional pathogenic and likely pathogenic variants in the adjacent sequenced regions.

### 3.4. Comparison of Two Genotyping Methods

The TaqMan custom panel included 116 assays for 115 variants in CFTR, PAH, SERPINA1, and GJB2 genes and was described earlier [8]. Genotyping using the TaqMan custom panel was performed in ESSE-Vologda and BCS samples. Genotyping in ESSE-Ivanovo and RPS-CP was performed using the NGS custom panel that included the same 115 variants in CFTR, PAH, SERPINA1, and GJB2 genes. Though NGS can identify all potentially pathogenic variants in selected genes, we limited the sequencing target to 115 variants included in the real-time PCR panel with 25 bp padding. This design provided a modest breadth of coverage of all coding exons for the targeted genes, namely 29% for CFTR, 15% for PAH, 12% for SERPINA1, and 22% in the case of GJB2. In the CFTR gene, 9 of 27 exons were not covered at all, 2 of 13 in PAH, 3 of 7 in SERPINA1. There is one exon in GJB2, and it was covered. In order to validate our results on data of all exon sequences in the studied genes, genotyping of all 115 variants and detection of other pathogenic variants were performed by exome sequencing for RPS-E (*n* = 127).

The verification of genotyping results for both genotyping methods was conducted by Sanger sequencing. The proportion of confirmed results was 86.67% for the TaqMan custom panel using QuantStudio 12 K Flex (Thermo Fisher Scientific, Waltham, MA, USA), 94.32% for the NGS custom panel and 89.36% for the exome sequencing using Nextseq 550 (Illumina, San Diego, CA, USA). The verification by Sanger sequencing confirmed more genotypes in the case of the NGS method. Fewer confirmed results for the TaqMan method can be explained by lack of positive controls for all alleles that are needed for better genotyping accuracy.

Cross-platform validation was performed using the NGS custom panel for genotyping 25 participants from ESSE-Vologda. The small size of the cross-validation group is due to financial reasons and is one of the limitations of our study. All nine observed variants were confirmed, no other variants included in the custom panel were found. Both methods detected a similar percentage of the participants who carried at least one variant: 14.63% for TaqMan and 15.71% and 13.22% for custom panel and exome sequencing, respectively (Table 2).

Unfortunately, both genotyping methods failed to achieve full genotyping precision. For example, analysis using the TaqMan custom panel did not reveal any carriers of the GJB2 variant rs80338939 (ESSE-Vologda and BCS), which is most common among Russian patients with SNHL [23,38]; thus, we suggested the incorrect work of the assay (assay ID ANEPWEH). This variant was detected using the NGS custom panel with AF of 1.74% (ESSE-Ivanovo). Another example of failed detection was rs397508184 (CFTR) using the NGS custom panel due to low coverage (ESSE-Ivanovo and RPS-CP). The validation of the results by exome sequencing showed two variants included in the custom panel that were uncovered in exomes: intronic rs75039782 (CFTR) and splice site rs80338940 (GJB2). 

Thus, by comparing genotyping results by QuantStudio 12 K Flex and Nextseq 550, we can conclude that, because of relatively lower cost, simple genotyping data analysis and easier detection of large indels are the main advantages of the TaqMan custom panel. However, the major advantages of the NGS custom panel are related to the better accuracy of the results as well as detection of additional pathogenic variants.

## 4. Discussion

In general, our data agree with previously published studies where differences between the Russian and European populations were shown for some rare variants [9,10]. It was shown previously that CFTRdele2,3 variant (hg19::chr7:117138367-117159446del) has Slavic origin with the highest frequency among CF patients of 6.4% in Czechia (5.8% in Russia) [39]. Similarly, it was reported that rs397508686 (*CFTR*) has a lower frequency worldwide and different frequencies in Russian regions, with the highest ones in the regions of the Middle Ural; up to 3.23% frequency among patients of Ural Federal District was reported in 2019, according to the Russian CF Patients Registry [40,41].

In addition, we compared our AF results to those in the known data for the Russian population, where available [6,10]. A total of six *CFTR* variants had AF for the Russian population (*n* = 1324) in the study by Petrova et al. [6]—rs121908751 (E92K), rs397508686 (L138ins), rs113993960 (F508del), rs121908776 (1677delTA), rs77010898 (W1282X), and hg19::chr7:117138367-117159446 (CFTRdele2,3). There were no statistically significant differences in AF compared to this study. Another research representing AF for the Russian population was performed by Barbitoff et al. [10]. The RUSeq database [42] contains data on AF for a total of 55 variants from our panel and for seven additionally detected variants. AF is presented for three distinct subgroups of samples. For AF comparison, the AF that most likely represents the European part of Russia was used [10]. As a result, AF for all variants were highly correlated.

According to the information obtained in our study, the choice of the most appropriate method for genotyping depends on the number of variants included in the study and economic limitations. In the case of testing for a small number of variants, the TaqMan method would certainly be more convenient, as it is faster, simpler, and relatively cheap [43]. The NGS method became the most suitable for the custom panels, including a high number of variants and, consequently, having a high detection rate [44]. Since the sequencing identified 13 extra pathogenic variants located in the targeted regions but not included explicitly in the custom panel, it is likely that for more effective carrier screening sequencing of all relevant exons with extra padding followed by interpretation based on the annotation and AF of discovered variants will be the most optimal solution [45].

## 5. Conclusions

This study provides new AF data for variants associated with CF, PKU, A1ATD, and SNHL as well as a review of genotyping methods that are usually used for carrier screening. The obtained results demonstrate differences in AF for 23 variants between the Russian population and NFE. We believe that our results will aid the future population carrier-screening programs.

## Figures and Tables

**Table 1:** Description of studied groups of samples.

Sample	Sample Description	Number of Participants Taken for AF Calculation	Genotyping Technology
ESSE-Ivanovo	representative sample of the Ivanovo region	1667	NGS
ESSE-Vologda	representative sample of the Vologda region	1243	real-time PCR
BCS	clinic visitors of reproductive age of the N.E. Bauman Scientific and educational medical-technological center	350	real-time PCR
RPS-CP	patients observed at the NMRC for Therapy and Preventive Medicine	440	NGS
RPS-E		121	NGS

BCS—Bauman Center sample; CP—custom panel sequencing; E—exome sequencing; ESSE—“Epidemiology of Cardiovascular Diseases and Risk Factors in Regions of the Russian Federation” study; NGS—next generation sequencing; NMRC—National Medical Research Center; PCR—polymerase chain reaction; RPS—Russian patients sample.

**Table 2:** The distribution of the detected variants from the custom panel in the studied groups of samples.

Parameters	ESSE-Vologda (*n* = 1243)	BCS (*n* = 350)	ESSE-Ivanovo (*n* = 1667)	RPS-CP (*n* = 440)	RPS-E (*n* = 121)	All (*n* = 3821)
Number of detected variants						
in *CFTR* (65 variants)	7	2	8	5	1	12
in *PAH* (23 variants)	7	4	9	7	1	13
in *SERPINA1* (10 variants)	3	2	4	3	2	4
in *GJB2* (17 variants)	3	4	7	7	4	9
overall (% of 115 variants)	20 (17.39)	12 (10.43)	28 (24.35)	22 (19.13)	8 (6.96)	38 (33.04)
additional pathogenic/likely pathogenic variants detected with NGS	-	-	7	4	3	13
Fraction of carriers, % (number of carriers) with variants						
in *CFTR*	2.82 (35)	1.71 (6)	1.68 (28)	1.36 (6)	0.83 (1)	1.99 (76)
in *PAH*	2.33 (29)	2.00 (7)	2.10 (35)	2.95 (13)	0.83 (1)	2.22 (85)
in *SERPINA1*	4.83 (60)	2.29 (8)	4.32 (72)	3.64 (16)	4.13 (5)	4.21 (161)
in *GJB2*	6.60 (82)	4.86 (17)	9.06 (151)	7.73 (34)	7.44 (9)	7.67 (293)
Fraction of participants who carried at least one variant, % (number of participants)	15.85 (197)	10.29 (36)	16.32 (272)	13.41 (59)	13.22 (16)	15.18 (580)

BCS—Bauman Center sample; CP—custom panel sequencing; E—exome sequencing; ESSE—“Epidemiology of Cardiovascular Diseases and Risk Factors in Regions of the Russian Federation” study; NGS—next generation sequencing; RPS—Russian patients sample.

**Table 3:** List of additional variants found in adjacent sequenced regions.

Variant	Reference/Alternative Allele	Gene	Consequence	Clinical Significance	Samples
rs374572413	C/T	*GJB2*	missense	likely pathogenic	RPS-CP
rs750188782	GCACACGTTCTTGCAGC/G	*GJB2*	frameshift	pathogenic	ESSE-Ivanovo
rs786204491	G/GT	*GJB2*	stop gained, frameshift	pathogenic	ESSE-Ivanovo
rs140175796	T/A	*PAH*	missense	likely pathogenic	RPS-E
rs199475679	C/T	*PAH*	missense	likely pathogenic	ESSE-Ivanovo, RPS-CP
rs199475696	A/G	*PAH*	missense	pathogenic	RPS-CP
rs5030856	T/C	*PAH*	missense	pathogenic	RPS-E
rs542645236	T/C	*PAH*	missense	pathogenic	RPS-CP
rs62507344	G/A	*PAH*	splice region, intron	pathogenic/likely pathogenic	ESSE-Ivanovo
rs62642939	C/T	*PAH*	missense	pathogenic	ESSE-Ivanovo
rs62642945	G/A	*PAH*	missense	likely pathogenic	ESSE-Ivanovo
rs62644469	A/G	*PAH*	missense	likely pathogenic	RPS-E
rs76687508	G/A	*PAH*	missense	pathogenic	ESSE-Ivanovo

BCS—Bauman Center sample; CP—custom panel sequencing; E—exome sequencing; ESSE—“Epidemiology of Cardiovascular Diseases and Risk Factors in Regions of the Russian Federation” study; RPS—Russian patients sample.

**Table 4:** Variants with significant differences in AF between studied samples and NFE data.

Variant	Reference/Alternative Allele	Gene	AF (gnomAD NFE)	AF ^1^	*p* Value	p adj ^2^	Samples	Number of Participants
rs397508612	GGT/G	*CFTR*	0	0.000132696	<0.001	<0.001	all	3768
CFTRdele2,3		*CFTR*	0.000052934	0.000859107	<0.001	<0.001	ESSE-Ivanovo, ESSE-Vologda	2910
rs113993960	ATCT/A	*CFTR*	0.0122683	0.006711409	<0.001	0.001	ESSE-Ivanovo, ESSE-Vologda	2831
rs121908793	G/T	*CFTR*	0.000008795	0.00035461	0.001	0.011	ESSE-Ivanovo, ESSE-Vologda	2820
rs397508686	C/CCTA	*CFTR*	0	0.001208564	<0.001	<0.001	ESSE-Ivanovo, ESSE-Vologda	2896
rs75039782	C/T	*CFTR*	0	0.000177494	<0.001	<0.001	ESSE-Ivanovo, ESSE-Vologda	2817
rs78655421	G/A	*CFTR*	0.00265245	0.000729395	0.002	0.019	ESSE-Ivanovo, ESSE-Vologda	2742
rs35887622	A/G	*GJB2*	0.0124173	0.023494605	<0.001	<0.001	ESSE-Ivanovo, ESSE-Vologda	2873
rs72474224	C/T	*GJB2*	0.00132572	0.005217391	<0.001	<0.001	ESSE-Ivanovo, ESSE-Vologda	2875
rs80338940	C/T	*GJB2*	0.000324886	0.001395187	0.001	0.007	ESSE-Ivanovo, ESSE-Vologda	2867
rs80338939	AC/A	*GJB2*	0.00919691	0.017396521	<0.001	<0.001	ESSE-Ivanovo	1667
rs5030850	G/A	*PAH*	0.000035201	0.000394529	0.003	0.020	all	3802
rs5030858	G/A	*PAH*	0.00147698	0.007473062	<0.001	<0.001	ESSE-Ivanovo, ESSE-Vologda	2877
rs62642934	T/C	*PAH*	0.000044025	0.000518672	0.002	0.019	ESSE-Ivanovo, ESSE-Vologda	2892
rs17580	T/A	*SERPINA1*	0.0365343	0.011088011	<0.001	<0.001	ESSE-Ivanovo, ESSE-Vologda	2886
rs28929474	C/T	*SERPINA1*	0.0184094	0.011034483	<0.001	<0.001	ESSE-Ivanovo, ESSE-Vologda	2900
rs28931570	G/A	*SERPINA1*	0.00204994	0.000172295	<0.001	0.002	ESSE-Ivanovo, ESSE-Vologda	2902

^1^ Combined AF for groups of samples. ^2^ The Benjamini–Hochberg adjustment was performed for the entire set of variants. AF—allele frequency; ESSE—“Epidemiology of Cardiovascular Diseases and Risk Factors in Regions of the Russian Federation” study; NFE—non-Finnish Europeans.

**Table 5:** Additional pathogenic and likely pathogenic variants in the adjacent sequenced regions detected in studied samples with significant differences in AF between studied samples and NFE data.

Variant	Reference/Alternative Allele	Gene	Clinical Significance	AF (gnomAD NFE)	AF ^1^	*p* Value	p adj ^2^	Samples	Number of Participants
rs374572413	C/T	*GJB2*	likely pathogenic	0	0.000237304	<0.001	<0.001	ESSE-Ivanovo, RPS-CP	2107
rs750188782	GCACACGTTCTTGCAGC/G	*GJB2*	pathogenic	0	0.00029994	<0.001	<0.001	ESSE-Ivanovo	1667
rs786204491	G/GT	*GJB2*	pathogenic	0.000026434	0.00059988	0.004	0.012	ESSE-Ivanovo	1667
rs140175796	T/A	*PAH*	likely pathogenic	0.000096693	0.004132231	0.023	0.046	RPS-E	121
rs62642939	C/T	*PAH*	pathogenic	0	0.00029994	<0.001	<0.001	ESSE-Ivanovo	1667
rs62644469	A/G	*PAH*	likely pathogenic	0	0.004132231	<0.001	<0.001	RPS-E	121

^1^ Combined AF for groups of samples. ^2^ The Benjamini–Hochberg adjustment was performed for the entire set of variants. AF—allele frequency; CP—custom panel sequencing; E—exome sequencing; ESSE—“Epidemiology of Cardiovascular Diseases and Risk Factors in Regions of the Russian Federation” study; NFE—non-Finnish Europeans.

## Data Availability

The data used and/or analyzed during the current study are available from the corresponding authors on reasonable request. Individual genotype information cannot be made available in order to protect participant privacy.

## Supplementary Materials

The following supporting information can be downloaded at: https://www.mdpi.com/article/10.3390/jpm12071132/s1, Table S1: List of 115 variants in the *CFTR*, *PAH*, *SERPINA1*, and *GJB2* genes, included in the custom panel; Table S2: List of 116 TaqMan assays, included in the custom panel; Table S3: Allele frequencies of 115 variants included in the custom panel for all studied groups of samples; Table S4: Comparison of allele frequencies of 115 variants between two population samples ESSE-Vologda and ESSE-Ivanovo; Table S5: List of detected homozygotes, compound heterozygotes, and heterozygotes; Table S6: Additional pathogenic or likely pathogenic variants in adjacent regions detected by NGS; Table S7: Comparison of allele frequencies of 115 variants between two population samples ESSE-Vologda and ESSE-Ivanovo and gnomAD non-Finnish Europeans; Table S8: Comparison of allele frequencies of 115 variants between all studied samples and gnomAD non-Finnish Europeans; Table S9: Comparison of allele frequencies of additional pathogenic variants between population sample ESSE-Ivanovo and gnomAD non-Finnish Europeans; Table S10: Comparison of allele frequencies of of additional pathogenic variants between all studied by NGS samples and gnomAD non-Finnish Europeans; Table S11: Comparison of allele frequencies of 115 variants between all studied samples and closely related population data; Table S12: Comparison of allele frequencies of additional pathogenic variants between all studied by NGS samples and closely related population data.
